# Non-V600 BRAF mutations recurrently found in lung cancer predict sensitivity to the combination of Trametinib and Dabrafenib

**DOI:** 10.18632/oncotarget.11635

**Published:** 2016-08-26

**Authors:** Amir Noeparast, Erik Teugels, Philippe Giron, Gil Verschelden, Sylvia De Brakeleer, Lore Decoster, Jacques De Grève

**Affiliations:** ^1^ Laboratory of Molecular Oncology and Department of Medical Oncology, Oncologisch Centrum, UZ Brussel, Vrije Universiteit Brussel, Brussels, Belgium

**Keywords:** non-V600 BRAF, lung cancer, impaired-kinase, Trametinib, Dabrafenib

## Abstract

Approximately half of BRAF-mutated Non-small cell lung cancers (NSCLCs) harbor a non-V600 BRAF mutation, accounting for ∼40,000 annual deaths worldwide. Recent studies have revealed the benefits of combined targeted therapy with a RAF-inhibitor (Dabrafenib) and a MEK-inhibitor (Trametinib) in treating V600 BRAF mutant cancers, including NSCLC. In contrast, sensitivity of non-V600 BRAF mutations to these inhibitors is not documented. Non-V600 mutations can either increase or impair BRAF kinase activity. However, impaired BRAF kinases can still activate the ERK pathway in a CRAF-dependent manner. Herein, beyond describing a cohort of BRAF mutant NSCLC patients and functionally analyzing 13 tumor-derived BRAF mutations, we demonstrate that both types of non-V600 BRAF mutations can be sensitive to clinically relevant doses of Dabrafenib and Trametinib in HEK293T cells, in lung epithelial cellular model (BEAS-2B) and in human cancer cell lines harboring non-V600 BRAF mutations. ERK activity induced by both types of these mutations is further reduced by combinatorial drug treatment. Moreover, the combination leads to more prolonged ERK inhibition and has anti-proliferative and pro-apoptotic effects in cells harboring both types of non-V600 BRAF mutations. This study provides a basis for the clinical exploration of non-V600 BRAF mutant lung cancers upon treatment with Trametinib and Dabrafenib.

## INTRODUCTION

BRAF mutations are found in ∼8% of human cancers and occur in 6–8% of non-small cell lung cancers (NSCLCs) [1, 2]. Although cancers such as melanoma predominantly have V600 BRAF mutations, non-V600 BRAF mutations are very common in NSCLC [2–6]. Lung cancers with a non-V600 BRAF mutation are predicted to account for approximately 40,000 annual deaths worldwide [2, 7].

V600 mutated BRAF has constitutively high kinase activity towards its downstream effector, mitogen/extracellular signal-regulated kinase (MEK), which in turn results in strong activation of extracellular-signal-regulated kinase (ERK) [8]. High kinase activity towards MEK due to V600 mutations has also been shown in non-cellular systems [6, 9, 10]. Some non-V600 BRAF mutations confer high kinase activity in cell-free assays, while other non-V600 mutations result in impaired kinase activity. However, kinase-impairing BRAF mutations still induce ERK pathway activation when wild-type CRAF, a hetero-dimerization partner of BRAF, is also present in the cell [9].

The ERK pathway is the major deregulated pathway associated with BRAF-mutated cancers. ERK pathway inhibition has been shown to have anti-proliferative effects in cells harboring both kinase-activating and impairing BRAF mutations [10–13].

Over the last decade, BRAF-targeted therapies and drug development have focused specifically on inhibition of V600E mutated BRAF, mainly due to the high proportion of V600E mutations in melanoma.

The need for potent and selective RAF inhibitors led to the development of type I RAF inhibitors such as Vemurafenib and Dabrafenib. In contrast to type II inhibitors, such as Sorafenib, these drugs inhibit RAF by binding to its active conformation. These drugs also inhibit the kinase activity of wild-type RAFs in cell-free assays [14–16]. However, type I RAF-inhibitors induce RAF dimerization in RAF wild-type cells in which RAF monomers are in an inactive state. RAF dimerization leads to transactivation and hyperactivation of the inhibitor-free RAF protomer, ultimately resulting in ERK pathway activation. This “paradoxical ERK activation” occurs with non-saturating doses of RAF inhibitors and is dependent on the presence of CRAF [17–20].

Importantly, paradoxical ERK activation is associated with manifestation of cutaneous squamous-cell carcinoma and keratoacanthoma in patients treated with RAF inhibitors [1, 17, 18]. Another challenge with RAF-inhibitor monotherapy is early adaptive insensitivity to these drugs. Early adaptive insensitivity to RAF inhibitors in V600E-mutated cells is characterized by a RAF inhibitor-induced shift from RAF monomeric to dimeric signaling, in parallel with relief of negative ERK feedback. Reactivated ERK has been shown to be MEK inhibitor sensitive [21–24]. In the long term, early adaptive insensitivity to RAF inhibitors can favor secondary and more permanent resistance mechanisms, such as CRAF overexpression or RAS mutations, leading to complete resistance to these drugs [21, 23, 25–27].

Despite initially promising clinical results in V600E BRAF-mutated melanoma, Vemurafenib and Dabrafenib monotherapies ultimately end with drug resistance and relapse of the cancer [27–30].

In melanoma patients harboring BRAF-V600 mutations, a combination of Dabrafenib with the allosteric MEK inhibitor Trametinib has been shown to improve overall survival and decrease the risk of adverse events related to paradoxical ERK activation when compared to Vemurafenib and Dabrafenib monotherapy, although ultimately combined therapy also ends with resistance [22–24, 27, 29, 31, 32].

The benefit of combined therapy with Dabrafenib and Trametinib has been demonstrated in V600 BRAF mutated melanoma, NSCLC and colorectal cancer (CRC). In contrast, non-V600 BRAF mutations have not been included in clinical trials with selective RAF and/or MEK inhibitors [29, 33–35]; this limitation could be due to the small proportion of patients harboring non-V600 BRAF mutations in melanoma. In addition, many non-V600 BRAF mutations are kinase-impaired and thus considered unattractive for RAF-targeted therapy.

As ERK activation in both classes of non-V600 BRAF mutations is RAF and MEK dependent and cells harboring such mutations have been shown to be addicted to ERK activity [10–13], we hypothesized that these mutations confer sensitivity to combined RAF and MEK targeting.

In the present study, we first describe a cohort of NSCLC patients identified with BRAF mutations. Then, we functionally analyze a set of 13 different BRAF mutations derived from this cohort and supplemented with mutations reported previously by others in NSCLC patients. Finally, we examine the effects of clinically relevant doses of Dabrafenib and/or Trametinib on HEK293T cells co-expressing mutant BRAFs with wt-CRAF and human cancer cell lines harboring non-V600 BRAF mutations.

## RESULTS

### Patient characteristics and BRAF mutation genotypes

A total of 229 NSCLC patients underwent molecular testing for the presence of EGFR, ERBB2, KRAS, NRAS, HRAS and BRAF mutations between January 2006 and December 2014.

**Figure 1:**
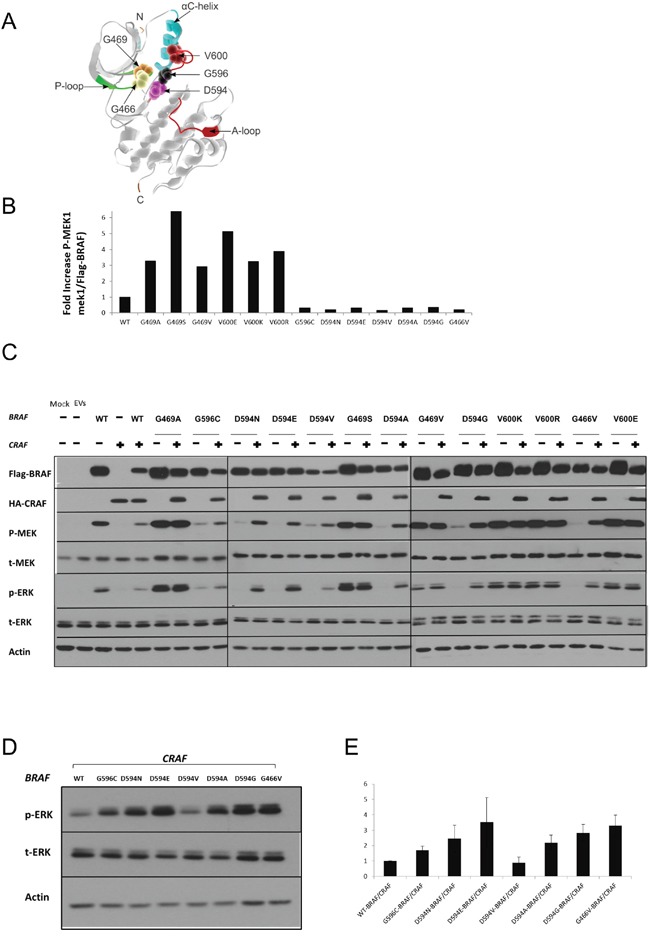
Characterization of BRAF mutations **A.** Different amino acid positions targeted by the BRAF mutations investigated in this study are mapped onto the 3D-Structure of the BRAF kinase-domain (PDB:4MBJ) [64], [P-loop: green; A-loop: magenta, alpha-C helix: cyan]. The 5 mutated positions are displayed as magnified van der Waals radii in distinctive colors. **B.** Equivalent amounts of BRAF proteins were subjected to *in vitro* kinase assays. For each type of recombinant BRAF protein, p-MEK and Flag-BRAF were quantified by ImageJ after western blotting. p-MEK levels were normalized to the corresponding Flag-BRAF levels, and fold increase over wild-type BRAF is displayed. **C.** HEK293T cells were transiently transfected with various BRAF expression vectors alone or co-transfected with a CRAF expression vector. Transfected cells were lysed 48 h post-transfection and subjected to western blot analysis to detect the indicated proteins. **D.** Comparative assay to determine the relative ERK activation induced by the different BRAF/CRAFs conferring impaired kinase activity. Only D594V BRAF/CRAF shows weaker ERK activity than wt-BRAF/CRAF. **E.** p-ERK and corresponding actin levels were quantified by Image J, p-ERK levels were normalized to the corresponding actin levels, and fold increase over wt-BRAF/CRAF is displayed. The Means ± sd are shown from three independent experiments. EV= Empty Vectors. When required, western blot bands were quantified by Image J.

Twelve patients (5.2%) were found to harbor seven different BRAF mutations. All the identified BRAF mutations were missense single nucleotide substitutions. Of the seven identified nucleotide substitutions, six were transversion events and 1 was a transition (Table 1, 2 & Figure 1A).

**Table 1:** Clinical characteristics of Belgian patients found with BRAF mutations

Patients	*BRAF* mutation	Nucleotide substitution	Age at diagnosis (years)	Survival (months)	Gender	Smoking history	TTF1	Stage	Firts-line Treatment
1	D594E	1782 T>G	47	9	female	non-smoker	-	NA	Cisplatin + Gemcitabine
2	G596C	1786 G>T	73	2	male	current	+	IV	NA
3	D594N	1780 G>A	77	8	male	current	NA	IV	Gemcitabine
4	D594N	1781 G>A	54	16	female	non-smoker	+	IV	Cisplatin + Gemcitabine
5	D594N	1782 G>A	NA	1	male	former	+	IV	Gemcitabine
6	G466V	1397 G>T	76	7	male	former	+	IV	Cisplatin + Gemcitabine
7	G469V	1406 G>T	70	2	female	former	+	IV	Carboplatin + Pemetrexed
8	G469A	1406 G>C	88	7	male	current	+	IV	NA
9	G469A	1406 G>C	69	NA	female	non-smoker	NA	IV	Cisplatin + etoposide
10	V600E	1799 T>A	56	9	female	former	+	IV	NA
11	V600E	1799 T>A	58	12	male	current	+	IV	Carboplatin + pemetrexed
12	V600E	1799 T>A	85	NA	female	NA	+	NA	NA

Abbreviations: TTF1, thyroid transcription factor; NA, not available.

**Table 2:** Summary of BRAF mutations characterization

Mutation	Exon	Position in kinase Domain	kinase activity towards MEK (kinase assay)	MEK/ERK activation (HEK293T)	Increased MEK/ERK in presence of CRAF (HEK293T)
**G466V**	11	p-loop	Impaired	No	Yes
**G469A**	11	p-loop	Active	Yes	No
G469S	11	p-loop	Active	Yes	No
**G469V**	11	p-loop	Active	Yes	No
D594A	15	DFG motif	Impaired	No	Yes
D594G	15	DFG motif	Impaired	No	Yes
**D594E**	15	DFG motif	Impaired	No	Yes
**D594N**	15	DFG motif	Impaired	No	Yes
D594V	15	DFG motif	Impaired	No	Yes (weak)
**G596C**	15	DFG motif	Impaired	No	Yes
**V600E**	15	A-loop	Active	Yes	No
V600K	15	A-loop	Active	Yes	No
V600R	15	A-loop	Active	Yes	No

Mutations in bold are found in the subset of Belgian NSCLC patients.

The mean age at the time of diagnosis for NSCLC patients with a BRAF mutation was 68.4 years (range between 47 and 88 years), which was comparable to the 72 years of the non-BRAF mutated patients. The gender ratio of BRAF-mutated NSCLC patients was 1:1. All patients identified with BRAF mutations had metastatic disease. Eight out of 12 NSCLC patients harboring a BRAF mutation were current or former smokers. The four BRAF-mutated NSCLC patients with a non-smoking history were female. Patient characteristics are summarized in Table 1. According to the patients' performance status and tolerance pattern, a platinum-based chemotherapy (cis or carboplatin) was chosen in combination with pemetrexed. Taking into account age, prognosis and comorbidity considerations, gemcitabine was administered instead of classical platinum-based schedules for some patients. The mean overall survival of the BRAF-mutated NSCLC patients was 7.4 months (range between 1 and 16 months, Supplementary Figure S1).

### Characterization of BRAF mutations

To characterize the seven NSCLC-derived BRAF mutations identified in our clinical samples and to test the sensitivity of a broader subset of non-V600 BRAF mutations to ERK pathway inhibitors, we generated 13 BRAF-expressing plasmids (in our cohort, supplemented by NSCLC mutations reported by others, recombinant BRAF proteins are 3X flag-tagged and are referred to as flag-BRAF for simplicity). Three of our mutations, D594E, D594N and G596C, were previously reported but not characterized. G469V was previously considered to be an activating mutation, without being characterized [36].

#### *In vitro* kinase assay

Recombinant BRAF mutant proteins were transiently expressed in HEK293T cells, purified by immunoprecipitation and quantified. Purified recombinant BRAF mutant proteins were subjected to *in vitro* kinase assays to determine their respective kinase activity towards the direct substrate of BRAF, MEK1 (Table 2 & Figure 1B).

Compared to wt-BRAF, the mutations G469A, G469S, G469V, V600E, V600K and V600R showed increased kinase activity towards kinase-dead MEK1. In contrast, G466V, D594A, D594G, D594E, D594N, D594V and G596C were kinase-impaired.

#### Kinase-active Mutant BRAF induces MEK/ERK pathway activation in HEK293T cells

To determine the impact of BRAF mutations on the MEK/ERK pathway in a cellular system, FLAG-tagged BRAF mutants were transiently expressed in HEK293T cells. Transfected cells were analyzed by western blot to determine the levels of phospho-MEK and phospho-ERK. Consistent with the results obtained in the *in-vitro* kinase assays, BRAF mutations G469A, G469S, G469V, V600E, V600K and V600R showed increased p-MEK and p-ERK levels compared to wt-BRAF. The BRAF mutants G466V, D594A, D594G, D594E, D594N, D594V and G596C did not induce MEK or ERK activation compared to wt-BRAF (Figure 1C & Table 2).

#### Co-expression of kinase-impaired BRAF and CRAF induces MEK/ERK pathway activation in HEK293T cells

As previously reported, kinase-impaired BRAF mutants can still activate the ERK pathway in a CRAF-dependent manner [9]. To further characterize our mutant BRAF constructs, we co-expressed each mutant BRAF with wt-CRAF in HEK293T cells.

BRAF mutants characterized as kinase-impaired in our *in vitro* kinase assay, clearly induced phosphorylation of MEK and ERK when co-expressed with CRAF (Figure 1, 1C & Table 2). Moreover, with the exception of D594V-BRAF/CRAF, higher p-ERK1/2 levels were observed in kinase-impaired-BRAF/CRAF co-transfectants compared to wt-BRAF/CRAF (Figure 1D & 1E).

### Effect of RAF/MEK/ERK pathway inhibitors on non-V600 BRAF mutant-induced ERK signaling

#### Inhibitory effect of Dabrafenib on mutant BRAF-induced ERK signaling

**Figure 2:**
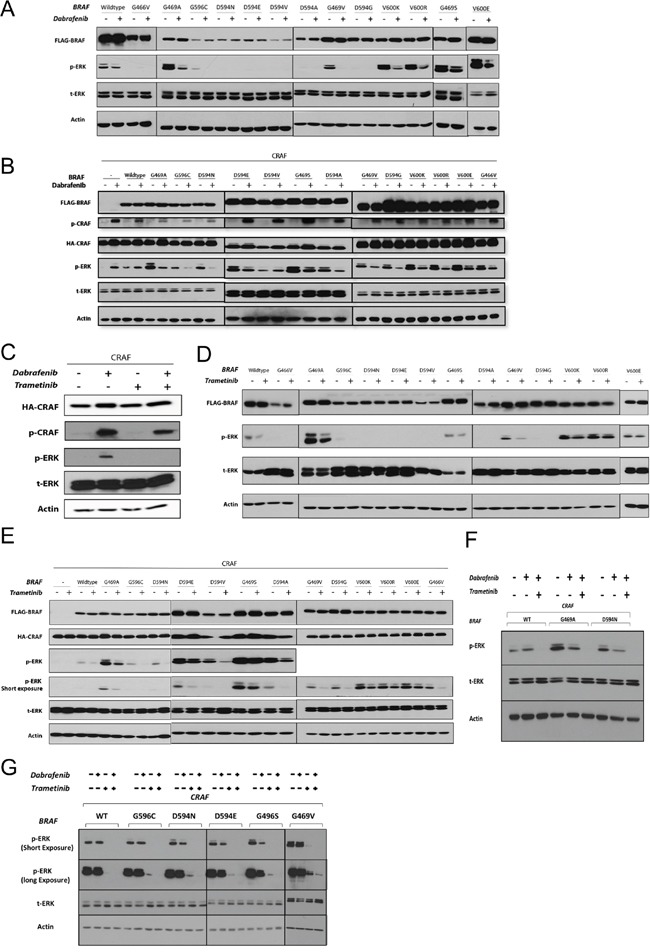
The effects of RAF and MEK inhibitors on non-V600 BRAF mutant-induced ERK signaling **A** & **B.** BRAF recombinant expression vectors were transfected singly (A) or co-transfected with CRAF (B) in HEK293T cells. After 48 h, cells were incubated for 2 h with the vehicle (DMSO) or Dabrafenib (2.5 μM). Whole cell lysates were subjected to western blot analysis for the indicated proteins. **C.** 48 h post-CRAF mono-transfection, HEK293T cells were treated with Dabrafenib (2.5 μM) in the presence or absence of Trametinib (25 nM) for 2 h (15 min Trametinib pre-treatment, 2 hr Trametinib monotherapy). **D** & **E.** Same as in (A & B) but treated with Trametinib (25 nM). **F.** Combined Dabrafenib and Trametinib treatment of the representative non-V600 BRAF co-transfectants (as in C); note that combined treatment strongly enhances ERK inhibition. **G.** BEAS-2B cells were co-transfected with each of the 5 previously uncharacterized BRAF mutations and CRAF. 48h post-transfection cells were incubated in conjunction with monotherapy or combined treatment with Dabrafenib (2.5 μM) and Trametinib (25 nM) for 2 h. Whole cell lysates were subjected to western blot analysis for the indicated proteins.

Among the clinically available RAF-inhibitors, Dabrafenib, whose primary target is V600E BRAF, has shown the highest affinity for CRAF in cell-free assays. The IC50 of Dabrafenib to inhibit CRAF is ∼10-fold less than that of Vemurafenib [14–16]. The average maximal plasma concentration of Dabrafenib in treated patients is between 1.5 μM and 2.8 μM [37]. To investigate the effect of Dabrafenib on ERK signaling induced by different BRAF mutations, we transiently expressed 13 BRAF mutants singly (Figure 2A) or with CRAF (Figure 2B) in HEK293T cells; we then evaluated the ERK activation status after 2 h of Dabrafenib treatment (2.5 μM).

As expected, Dabrafenib treatment of HEK293T cells singly expressing BRAF mutants with elevated kinase activity led to decreased p-ERK1/2 levels (Figure 2A).

Dabrafenib treatment of HEK293T cells co-expressing BRAF mutants (conferring elevated and impaired kinase activity) with CRAF resulted in decreased p-ERK1/2 levels (Figure 2B). The only exception was the impaired kinase BRAF mutant D594V, which showed increased p-ERK levels upon Dabrafenib treatment (Figure 2B). A similar increase in p-ERK levels was observed after Dabrafenib treatment of HEK293T cells transfected singly with CRAF or co-transfected with CRAF and wt-BRAF. The ERK-inhibitory inhibitory effect of Dabrafenib on G469S BRAF expressing cells was subtle.

Both classes of RAF inhibitors can induce phosphorylation of CRAF at serine 338 (S338), the regulatory phosphorylation site of CRAF protein [18, 38]. We observed that Dabrafenib treatment of all HEK293T transfectants resulted in phosphorylation of the transfected CRAF molecule at S338, irrespective of the presence and type of BRAF molecule (Figure 2B).

#### Inhibitory effect of Trametinib on mutant BRAF-induced ERK signaling

A recent study revealed the superior efficacy of Trametinib versus several other MEK-inhibitors in cells with KRAS mutations and CRAF-mediated ERK pathway activation [39]. This effect may potentially privilege Trametinib in targeting cells harboring kinase-impaired BRAF mutations, as these mutations also activate the ERK pathway in a CRAF-dependent manner. Trametinib has previously shown efficacy in cells with V600E/K BRAF mutations *in vitro* and in the clinic. Therefore, we chose to test the effect of Trametinib in the context of mutant BRAF signaling.

We first evaluated whether Trametinib induces paradoxical ERK activation or phosphorylation of CRAF at S338, as was observed with Dabrafenib, in CRAF-overexpressing monotransfectants. Trametinib treatment did not result in either ERK activation or CRAF phosphorylation (Figure 2C). In addition, combined Dabrafenib and Trametinib treatment inhibited Dabrafenib-induced ERK activation in CRAF-overexpressing monotransfectants (Figure 2C).

Trametinib treatment of HEK293T cells singly expressing BRAF mutations conferring elevated kinase activity, or co-expressing them with CRAF, resulted in decreased p-ERK1/2 levels. The ERK-inhibitory effect of Trametinib on HEK293T cells expressing G469S-BRAF was weak (Figure 2, 2D & 2E). Trametinib also decreased p-ERK levels in all the kinase-impaired BRAF/CRAF co-transfectants (Figure 2E).

#### Combinatorial Trametinib and Dabrafenib treatment enhances ERK inhibition in HEK293T and lung epithelial co-transfectants

To determine whether the combination of Trametinib and Dabrafenib enhances ERK inhibition, we investigated ERK inhibition by Dabrafenib in three representative co-transfectants (wt-BRAF/CRAF, G469A/CRAF and D594N/CRAF) in the presence or absence of Trametinib (Figure 2F). In all co-transfectants, combined treatment significantly enhanced ERK inhibition compared with Dabrafenib monotherapy. In addition we tested the effect of Dabrafenib and/or Trametinib in lung epithelial cells (BEAS-2B) co-transfected with 5 previously uncharacterized BRAF mutations and CRAF (Figure 2G). Dabrafenib monotherapy had only very weak ERK inhibitory effect. Trametinib single treatment caused strong ERK-inhibitory effect compared to Dabrafenib monotherapy whereas combinatorial treatment showed superior ERK-inhibitory effect compared to each of the single agents (Figure 2G). Combinatorial drug treatment also led to decreased viability in BEAS-2B cells stably transfected with two representative BRAF mutations (Supplementary Figure S2).

### Effect of Dabrafenib, Trametinib and their combination on non-V600 mutated BRAF human cancer cell lines

The non-V600 BRAF mutations that we tested in our HEK293T and BEAS-2B model are all clustered, either in the P-loop (ATP-binding pocket) or in the DFG motif (a.a. 594–596 within the activation segment) of the BRAF kinase domain. Unfortunately, there is no available BRAF mutant NSCLC cell line harboring a mutation within the DFG motif. Therefore, we analyzed two NSCLC adenocarcinoma cell lines harboring two different mutations along the P-loop of the BRAF kinase domain and a CRC cell line of adenocarcinoma origin with a BRAF mutation positioned within the DFG motif.

H1395 and H1666 are NSCLC cells harboring a homozygous G469A BRAF mutation (high kinase) and a heterozygous G466V BRAF mutation (impaired kinase), respectively. H508 is a CRC (caecal) adenocarcinoma cell line harboring a heterozygous G596R BRAF mutation (impaired kinase).

#### Combinatorial Dabrafenib and Trametinib treatment enhances their anti-proliferative effects in non-V600 BRAF mutated cell lines

Non-V600 BRAF-mutated cell lines were exposed to single agent monotherapy and a combination of Dabrafenib and Trametinib, for three or five days depending on the cell line and then subjected to a CellTiter-Glo assay to quantify cell viability.

**Figure 3:**
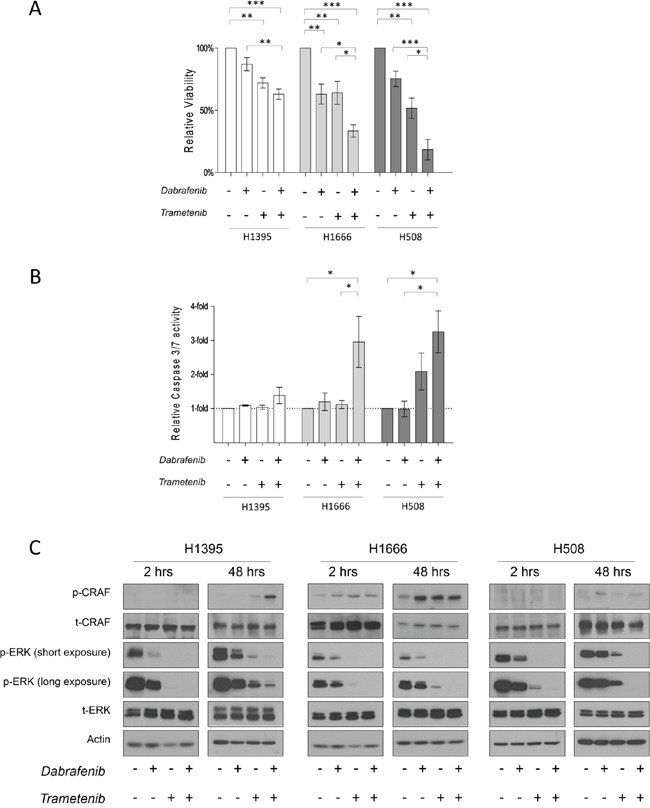
The effects of Dabrafenib, Trametinib and their combination on non-V600 BRAF mutated cell lines **A.** Cells were incubated for 3 days (H1395 & H1666) or 5 days (H508) with monotherapy or combined treatment with Dabrafenib (2.5 μM) and Trametinib (25 nM). Viability was measured, and relative viability was determined by normalizing to the vehicle group. Means ± SEM are from at least three independent experiments, each performed in quadruplicate. **B.** Cells were incubated for 3 days with monotherapy or combined treatment with Dabrafenib (2.5 μM) and Trametinib (25 nM). Caspase-3/7 activity was measured and normalized to the number of viable cells. Values are displayed as fold increase compared to the vehicle group. Means ± SEM are shown from at least three independent experiments, each performed in quadruplicate. **C.** Cells were incubated in conjunction with monotherapy or combined treatment with Dabrafenib (2.5 μM) and Trametinib (25 nM) for 2 h and 48 h. Whole cell lysates were subjected to western blot analysis for the indicated proteins. *p ≤ 0.05, * * p ≤ 0.01, * * * p ≤ 0.001.

Trametinib monotherapy had a stronger anti-proliferative effect than Dabrafenib on H1395 and H508 cells, while both monotherapies showed comparable anti-proliferative effects in H1666 cells (Figure 3A).

In all tested cell lines, combinatorial treatment led to enhanced anti-proliferative effects compared to monotherapy treatments and the vehicle control. Interestingly, stronger growth inhibition was observed in kinase-impaired BRAF-harboring cells. Compared to the vehicle control, H508, H1666 and H1395 cells showed an 82 ± 8.1%, 67 ± 4.9% and 37 ± 4.1% reduction in the number of viable cells, respectively, as a result of combined treatment with Dabrafenib and Trametinib.

To determine whether single or combined treatment triggered apoptosis, we measured caspase3/7 activation 72 h post drug treatment (caspase 3/7 glo test). We did not detect significant caspase 3/7 activation after 72 h of drug treatment in H1395 cells (Figure 3B). Upon single inhibitor monotherapy, increased caspase 3/7 activity was observed only in H508 cells treated with Trametinib.

In cell lines with impaired kinase BRAF mutations (H1666 and H508), combinatorial therapy induced a 3- and 4-fold increase, respectively, in caspase 3/7 activity compared with the vehicle control (Figure 3B).

#### Combination of Dabrafenib and Trametinib enhances and prolongs ERK inhibition in non-V600 BRAF mutated cell lines

We examined the acute and chronic effects of isolated and combined Dabrafenib and Trametinib treatment on the ERK pathway. In the given cell lines, we performed western-blot analysis on total lysates after 2 h and 48 h of drug treatment. All cell lines showed decreased p-ERK levels after 2 h of monotherapy treatment. Trametinib induced stronger ERK inhibition compared to Dabrafenib in all three cell lines. As expected, combined treatment caused the strongest ERK inhibition in all three cell lines (Figure 3C).

To determine whether combined treatment could overcome early adaptive insensitivity to Dabrafenib or Trametinib, we analyzed cell lysates 48 h post drug treatment. No rebound in p-ERK levels was detected in H1666 and H508 cells after 48 h of combined treatment, as opposed to treatment with single inhibitors. In H1395 cells after 48 h of combined treatment, a small increase in p-ERK levels was detected.

## DISCUSSION

In this study, we functionally analyzed a set of NSCLC-derived mutations clustered in exons 11 and 15 of the BRAF gene. These mutations were identified during routine diagnostic screening of a cohort of patients diagnosed and treated at our institution (seven mutations) or previously reported in the literature (six mutations). The seven mutations found at our institution were identified in a cohort of NSCLC patients preselected as having a limited or non-smoking history. Twelve out of the 229 NSCLC patients in the total cohort (5.2%) harbored a BRAF mutation. In contrast to melanoma, only 25% of the BRAF mutant NSCLC cases carried a mutation that involved the valine residue at position 600. Similar observations have been previously reported in NSCLC [3–6]. However, to our knowledge, our cohort of BRAF mutant NSCLC patients exhibits the highest frequency of non-V600 BRAF mutations ever reported in NSCLC. These BRAF mutant NSCLCs did not harbor concomitant KRAS, NRAS, EGFR, ERBB2 or ALK mutations.

Characterization of the BRAF mutants G469S, G469V, D594E, D594N and G596C is, to our knowledge, unprecedented. *In vitro* kinase assays showed that D594E, D594N and G596C result in impaired kinase activity, similar to previously characterized mutations clustered along the DFG motif.

In contrast, G469S and G469V, both positioned along the P-loop, exhibited higher kinase activities compared to wt-BRAF. The kinase activity of G469S was comparable to that of V600E.

Observations made with the *in vitro* kinase assays were confirmed in a HEK293T cell model.

Cells transfected with BRAF mutants with elevated kinase activity had increased p-MEK and p-ERK levels compared to those transfected with wt-BRAF, while BRAF mutants with impaired kinase activity showed decreased levels of p-MEK and p-ERK.

Impaired kinase BRAF mutations are known to dimerize with CRAF and allosterically transactivate it, leading to ERK pathway activation [40]. Therefore, we evaluated the impact of various BRAF mutations on ERK signaling in the presence of CRAF. Co-expression of BRAF mutants with impaired kinase activity and CRAF strongly increased MEK and ERK phosphorylation. ERK activity in HEK293 cells co-expressing BRAF mutants with impaired kinase activity and CRAF was higher compared to that in wt-BRAF/CRAF co-transfectants. The only exception was the D594V BRAF mutant, as its co-expression with CRAF resulted in a modest increase in MEK and ERK activity. This result was consistent with that of a previous report in another cellular model [9]. These results suggest that D594V BRAF may be a non-pathogenic variant or a kinase-independent oncogene. While the role of CRAF in NSCLC has not been fully elucidated, CRAF overexpression has been reported in a large subset of lung cancers [8, 41, 42]. In a transgenic mouse model, lung-targeted overexpression of CRAF can induce development of NSCLC [43]. In cancers with KRAS mutations, CRAF plays a pivotal role in ERK signaling and in the early stages of oncogenesis [44, 45]. CRAF knockdown has been shown to inhibit growth of NSCLC cells harboring BRAF mutations conferring impaired kinase activity [10].

A clinically relevant dose of Dabrafenib, a type I RAF-inhibitor, in HEK293T cells reduced ERK activity induced by BRAF mutations conferring high kinase activity in the presence and absence of CRAF. Furthermore, Dabrafenib also inhibited ERK activity induced by BRAF mutants conferring impaired kinase activity co-expressed with CRAF. The only exception was once again the D594V BRAF/CRAF co-transfectant, which showed increased ERK activity upon Dabrafenib treatment (similar to wt-BRAF/CRAF). When RAFs are in an inactive state, RAF inhibitors can induce dimerization of inhibitor-bound RAF with inhibitor-free RAF causing its transactivation and hyperactivation, ultimately leading to ERK pathway activation [19]. As we observed, D594V BRAF, even in the presence of CRAF, did not induce strong ERK activity. This result indicates that RAF isoforms remain inactive in the presence of the D594V BRAF. Therefore, pathway activation upon Dabrafenib treatment may be explained by the opposing mode of action of Dabrafenib towards the inactive fraction of RAF isoforms.

Dabrafenib-induced paradoxical ERK activation was observed in cells singly expressing exogenous CRAF, as well as in cells co-expressing exogenous wt-BRAF and CRAF. This result is consistent with reports on the CRAF dependence of paradoxical ERK activation induced by RAF inhibitors. [17–19, 46].

Unlike Dabrafenib, the MEK-inhibitor Trametinib did not induce ERK activation in CRAF mono-transfectants. In HEK293T cells, Trametinib modestly decreased ERK activity induced by BRAF mutations conferring elevated kinase activity while strongly inhibited kinase-impaired BRAF/CRAF-induced ERK activation. Moreover, we showed that combinatorial treatment with Trametinib and Dabrafenib overcame paradoxical ERK activation in wt-BRAF/CRAF HEK293T and lung epithelial co-transfectants and substantially improved ERK inhibition in non-V600 BRAF mutants with both impaired and elevated kinase activity.

Serine-338 is a regulatory site in the N-region of CRAF whose phosphorylation is believed to be a preliminary step for CRAF activation. However, the exact mechanism and full sequence of events prior and subsequent to CRAF activation remain largely unknown [47, 48]. Both classes of RAF-inhibitors have been shown to induce phosphorylation of CRAF at S338 [18, 19]. We also observed the similar phenomenon with Dabrafenib.

Notably, activated CRAF is suggested to be associated with several kinase-independent functions related to oncogenesis [49–53]. Therefore, it has yet to be determined whether over time activated CRAF can contribute to kinase-independent resistance mechanisms to RAF inhibitors in BRAF mutated cancers [54].

Using our mutant BRAF/CRAF co-expressing HEK293T model and lung epithelial cells, we demonstrated that non-V600 BRAF mutations, including kinase-impaired mutations, are “druggable”.

To evaluate whether these results could be confirmed in human cancer cell lines, we tested the effects of Dabrafenib, Trametinib and their combination on cell lines harboring BRAF mutations located at positions other than V600, which were representative of distinctive positions along the BRAF kinase domain. As there is no NSCLC cell line with a BRAF mutation in the DFG motif, we included a CRC cell line of adenocarcinoma origin harboring a BRAF mutation along this motif, although we are aware that the significance of BRAF mutations and their inhibition might be context dependent. Concerning the five mutations of which the characterization is unprecedented, in the future the availability of patient-derived NSCLC cell lines harboring these BRAF mutations would help broaden our knowledge regarding mechanism of oncogenesis and drug response.

The combination of Dabrafenib and Trametinib resulted in enhanced growth inhibition compared to monotherapy treatments in the three tested cell lines.

Combined treatment resulted in significantly enhanced caspase 3/7 activity in H1666 and H508 cells but not in the H1395 cell line. This observation is consistent with a previous report on a different MEK inhibitor (PD0325901) that inhibits H1395 cell growth without inducing apoptosis [55]. However, in previous studies, another NSCLC-derived cell line harboring the same BRAF mutation (G469A) showed both growth inhibition and apoptosis upon MEK inhibition [55, 56]. Therefore, this lack of apoptosis induction by MEK inhibitors in H1395 cells seems to be related to unknown cellular or genetic factors, while a potential therapeutic benefit might still be derived from the growth inhibition.

In all the tested cell lines, combined treatment enhanced and prolonged ERK inhibition compared to monotherapy treatment (48 h). As in V600-mutated cells, this result suggests that combined RAF and MEK targeting can overcome the early adaptive insensitivity to RAF inhibitor monotherapy in non-V600 BRAF mutated cells.

Interestingly, H1666 and H508 cell lines were previously shown to be resistant—or poorly responsive—to the selective RAF-inhibitor Vemurafenib, as well as to the MEK inhibitor Selumetinib [57–60]. As ERK activation in the given cell lines is CRAF-mediated, the fact that Vemurafenib is a weak CRAF inhibitor may contribute to the differential responses. Notably, CRAF knockdown has been shown to inhibit growth of H1666 cells [10].

A recent study investigated the differential efficacy of MEK inhibitors in KRAS-mutated cancer cells in which ERK pathway activation is supposed to be CRAF-mediated. The study reveals that two MEK inhibitors (Selumetinib and PD0325901) are less efficient than Trametinib for sustained suppression of CRAF-mediated ERK activity. This result may explain why H1666 and H508 cells are more sensitive to Trametinib than to Selumetinib, as BRAF mutations in these cell lines activate the ERK pathway in a CRAF-dependent manner, similarly to KRAS-mutated cells. In contrast, H1666 was previously reported to be sensitive to PD0325901, which is mechanistically similar to Selumetinib, by means of making inhibited MEK prone to reactivation by CRAF [39, 61]. Therefore, further investigation of the comparative efficacy of various MEK inhibitors in BRAF-mutated cancers would help to explain these contradicting results.

Due to the small size of our patient cohort, we could not detect any significant clinical differences between BRAF mutated and other molecular subtypes of lung cancer or between different BRAF mutation types.

In conclusion, our findings confirm that non-V600 BRAF mutations are not rare in NSCLC. We demonstrate that non-V600 BRAF mutations, resulting in either high or impaired kinase activity, confer sensitivity to combined Dabrafenib and Trametinib treatment. Dabrafenib monotherapy has only a weak effect, and targeting impaired-kinase BRAF mutations or CRAF-overexpressing cells with Dabrafenib alone may even lead to paradoxical ERK activation. Notably, the sensitivity of cells with mutations conferring impaired kinase activity to combinatorial Dabrafenib and Trametinib treatment has not previously been described. Our findings support the clinical exploration of the efficacy of combined Dabrafenib-Trametinib treatment in advanced NSCLC patients harboring non-V600 BRAF mutations in their tumors.

## MATERIALS AND METHODS

### Patient cohort

Patients with locally advanced or metastatic (stage IIIB or stage IV) adenocarcinoma of the lung and a non- or limited-smoking history were included in the study.

Prospectively collected data included age at diagnosis, gender, smoking history, ethnicity, Karnofsky performance status scale (KPSS), treatments and survival time.

### Diagnostics and mutation analysis

DNA was isolated from formalin fixed paraffin embedded (FFPE) tumor samples (Qiagen 56404). The presence of EGFR, ERBB2, KRAS, NRAS, HRAS and BRAF mutations was tested using denaturing gradient gel electrophoresis [62] or next generation sequencing-based methods.

### DNA constructs

A full-length BRAF cDNA-bearing cassette was a kind gift from Loredana Vecchione in Prof. Sabine Tejpar's lab (Catholic University Leuven). The BRAF coding sequence was PCR-cloned (AccuPrime, Life Technologies, 12344-024) into the desired destination vector, PX3FLAG-CMV-14 (Sigma, E7908). An empty vector (puno1-hRAF1) and HA-tagged-CRAF expression vector (customized) were obtained from InvivoGen (Toulouse, France).

### Site-directed mutagenesis

Desired mutations were introduced in the BRAF coding sequence using site-directed mutagenesis (GeneArt Site-Directed Mutagenesis System, Life-Technologies, A13312). The full length BRAF coding sequence and the insertion sites in the expression vector were fully sequenced for each construct generated.

### Cell culture and transfections

HEK293T cells were a kind gift from Ron Kooijman (FARC, Vrije Universiteit Brussel). Cells were cultured in Dulbecco's Modified Eagles Medium (DMEM) (Life Technologies, 31966-047) supplemented with 10% fetal bovine serum (FBS) (Perbio Science, SV30160.03) and penicillin-streptomycin (pen-strep) (Life Technologies, 15140-148). Cells were passaged every three days.

Prior to transfection, 150,000 cells/well were seeded in 24-well plates and incubated overnight (in antibiotic-free medium). The following day, the medium was changed to OptiMEM (Life Technologies 31985-047) and retained in the incubator for 30 minutes. Transfections and co-transfections were performed using Lipofectamine-2000 (116680-19) according to the manufacturer's instructions. Six hours post-transfection, OptiMEM was changed to antibiotic-free DMEM (supplemented with 10% FBS). Cells were collected 48 h post-transfection for western blot analysis.

BEAS-2B cells were kind gift from Prof. Didier Cataldo (University of liége) and were cultured in DMEM supplemented with 10% (FBS) and pen-strep. BEAS-2B cells were transfected as described for HEK293T cells, due to increased toxicity the amount of DNA transfected was reduced to 0.4μg.

Stably transfected BEAS-2B lines were generated by reseeding the transiently transfected cells (after 48h) in presence of corresponding antibiotics for 2-3 weeks. Cells transfected with BRAF expression cassettes or PX3FLAG-CMV-14 vector were selected in medium supplemented with 750 μg/ml G418 (sigma-aldrich), and cells co-transfected (in two steps) with CRAF expression cassette or puno1-hRAF1 were selected in medium supplemented with 750 μg/ml G418 and 5 μg/ml Blasticidin (invivogen).

H1395, H1666 and H508 cells were obtained from ATCC. H1395 and H508 were cultured in RMPI-1640 (ATCC: 30-2001) supplemented with 10% FBS and pen-strep. For H1666 cells, F12-based (ATCC: 30-2006) ACL-4 medium was freshly prepared following ATCC's recommendations. All cells were periodically tested for mycoplasma.

### *In vitro* kinase assay

BRAF proteins were transiently expressed in HEK293T cells and subsequently purified using a flag-immunoprecipitation kit (Sigma, FLAGIPT1). Equivalent amounts of BRAF proteins were subjected to a kinase reaction together with kinase-dead MEK1 substrate (100 ng BRAF kinase+500 ng inactive MEK1, as recommended by Merck Millipore, 14-420) in the presence of 10 mM ATP (Ultra Pure ATP, Promega) at 30°C. Kinase reactions were terminated after 30 minutes, and reaction solutions were visualized by chemiluminescence using anti-phospho-MEK and anti-Flag antibodies. ImageJ software (http://imagej.nih.gov/ij/) was used to quantify p-MEK and flag-BRAF levels. p-MEK levels were normalized to the related Flag-BRAF levels. Fold increase over wt-BRAF was calculated and reported.

### Western blot

Cells were lysed in 1% triton-X buffer supplemented with phosphatase, protease inhibitor (Sigma, P8340 and P5726) and leupeptin trifluoroacetate (Sigma, L2023).

Lysates were centrifuged, and protein concentration was determined using the Bradford protein assay kit (Bio-Rad). Equivalent amounts of protein were loaded on a 10% resolving acrylamide gel. Protein transfer was conducted overnight at 4°C using polyvinylidene fluoride membranes (PVDF). The membranes were blocked with 5% non-fat milk and washed. Blocked membranes were labeled with primary antibody overnight at 4°C followed by 1 h incubation with the corresponding secondary horseradish peroxidase (HRP)-conjugated antibody at 37°C. Detection was performed using enhanced chemiluminescence (ECL) detection reagent (Isogen Life Science, K-12045-D20) and Fuji super films (104253) or by ImageQuant LAS-4000 (GE Healthcare). Western blot antibodies were: phospho-MEK1/2 (cell signaling, 9121), total-MEK1/2 (cell signaling, 9122), phospho-ERK1/2 (cell signaling, 4370), total ERK1/2 (cell signaling, 4695), phospho-S338-CRAF (cell signaling, 9427), HA-TAG (cell signaling, 2367), FLAG (Sigma, F1804), and anti-beta ACTIN (Sigma, A1978).

### Inhibitors

Dabrafenib (TAFINLAR) was provided by GlaxoSmithKline. Trametinib (GSK1120212) was obtained from selleckchem (S2673).

### Cell viability and caspase 3/7 activity assays

Viability was determined using a CellTiter-Glo Luminescent kit (Promega: G7570). Caspase 3/7 activity was determined with a Caspase-Glo 3/7 Assay Kit (Promega; G8091), as previously described [63]. Briefly, for both assays, cells were seeded in 384-well plates at 800–2,500 cells/well. The following day, drugs were added (with equal amounts of dimethyl sulfoxide (DMSO) for all conditions) and incubated until the designated time points for each assay. Caspase activities were measured and normalized to the number of viable cells from the corresponding wells in the same plates.

### Statistical methods

Fisher's exact test was used to compare the gender and smoking history between V600 and non-V600 patients as well as between patients with BRAF containing mutations conferring high and impaired kinase activity. A Mann-Whitney U test was used to compare age at diagnosis between the given groups. Overall survival was estimated using the Kaplan-Meier method, and the curves were compared using log-rank tests. For proliferation and caspase 3/7 data, means of at least 3 independent experiments, each performed in quadruplicate, were compared by a one-way Anova, post-hoc test. SPSS was used for statistical analysis and generation of the Kaplan-Meier curves.

## SUPPLEMENTARY MATERIALS FIGURES


