# Supplementary material for: Non-V600 BRAF mutations recurrently found in lung cancer predict sensitivity to the combination of Trametinib and Dabrafenib
Source: Oncotarget. 2016 Aug 26;8(36):60094–108. doi: 10.18632/oncotarget.11635 (PMC5601124; doi:10.18632/oncotarget.11635)
Supplement: Supplementary file 1 [file oncotarget-08-60094-s001.pdf]

Non-V600 BRAF mutations recurrently found in lung cancer predict sensitivity to the combination of Trametinib and Dabrafenib

SUPPLEMENTARY FIGURES

S1A

Overall Survival of BRAF-mutated patients

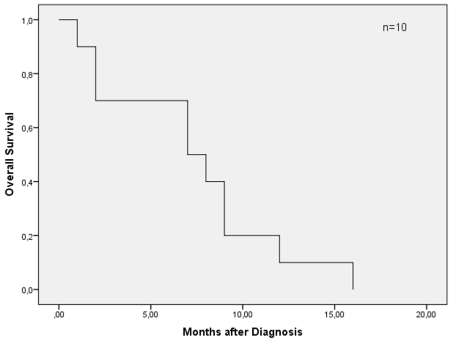

S1B

Overall Survival of patients with Impaired-Kinase versus High-Kinase BRAF mutations

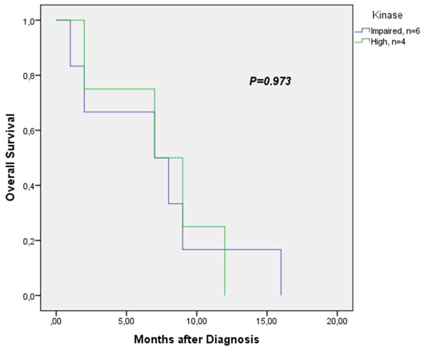

S1C

Overall Survival of patients with Non-V600 versus V600- BRAF mutations

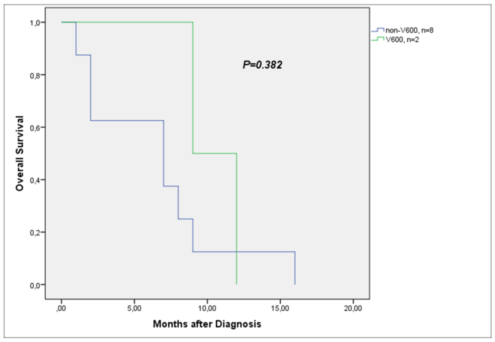

Supplementary Figure S1: Kaplan-Meier survival curves were generated using SPSS. A. Overall survival of BRAF-mutated NSCLC patients **B.** Overall survival of patients with Impaired-Kinase BRAF was compared to those with High-kinase BRAF mutations **C.** Overall survival of patients with V600 BRAF was compared to those with non-V600 BRAF mutations. A log-rank test revealed no significant differences between the compared groups.

S2A

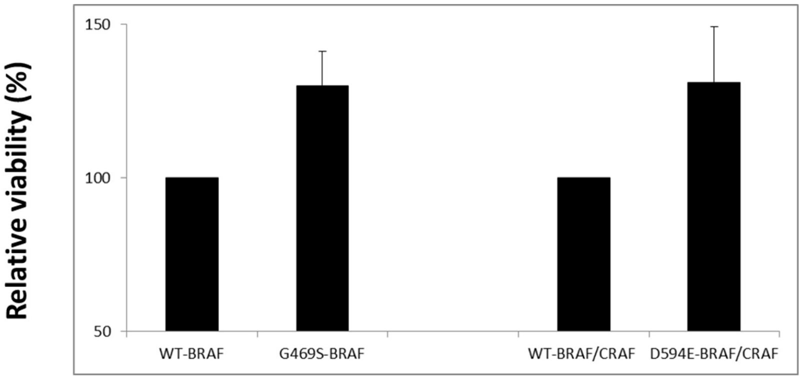

S2B

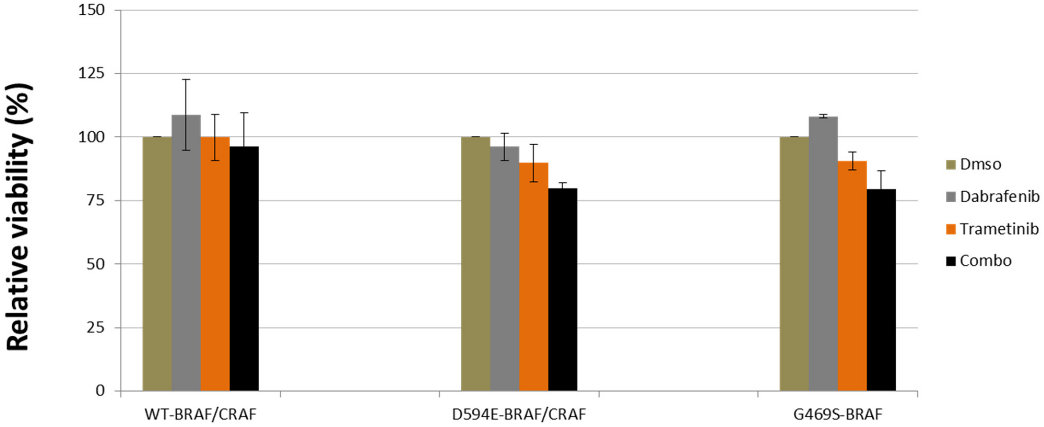

S2C

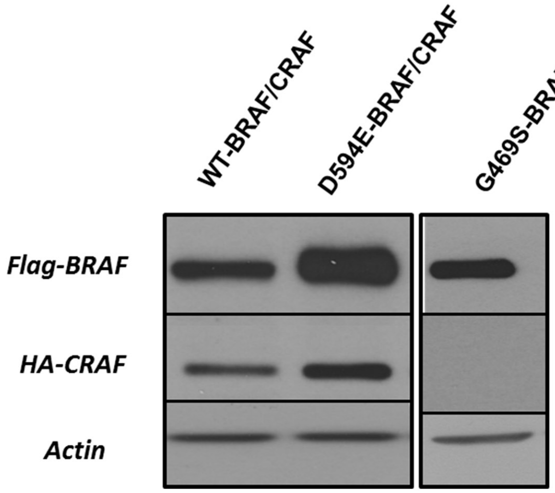

Supplementary Figure S2: The effects of Dabrafenib, Trametinib and their combination on stably transfected BEAS-2B cells. A. Both G469S-BRAFand D594E-BRAF/CRAF stable transfectants showed increased viability compared to respectively WT-BRAF (30 ± 11%) and WT-BRAF/CRAF (31 ± 18%) stable-transfectants. However as wild-type and mutant recombinant genes are expressed at significantly different levels in the transfected clones (S.2 C), one cannot exclude that some of the differences in viability could be due to different expression levels of recombinant proteins (wild-type vs. mutant). **B.** Cells were incubated for 2 days with monotherapy or combined treatment with Dabrafenib (2.5 μM) and Trametinib (25 nM). Viability was measured, and relative viability was determined by normalizing to the vehicle group. Means ± SEM are from two independent experiments, each performed in quadruplicate. **C.** BEAS-2B stable transfectants were lysed and subjected to western blot analysis to determine the expression of the transfected genes.
